# An SEIR model with infected immigrants and recovered emigrants

**DOI:** 10.1186/s13662-021-03488-5

**Published:** 2021-07-16

**Authors:** Peter J. Witbooi

**Affiliations:** Department of Mathematics and Applied Mathematics, University of the Western Cape, Robert Sobukwe Rd, Bellville, 7530 South Africa

**Keywords:** 92D30, 34K20, Basic reproduction number, Stable equilibrium, Imported infection, Recovered emigrant, Measles

## Abstract

We present a deterministic SEIR model of the said form. The population in point can be considered as consisting of a local population together with a migrant subpopulation. The migrants come into the local population for a short stay. In particular, the model allows for a constant inflow of individuals into different classes and constant outflow of individuals from the R-class. The system of ordinary differential equations has positive solutions and the infected classes remain above specified threshold levels. The equilibrium points are shown to be asymptotically stable. The utility of the model is demonstrated by way of an application to measles.

## Introduction

The fight against infectious diseases presents a major challenge to nations and a huge financial burden for governments. In recent months due to the emergence of COVID-19, there has been much publicity in the media around the role of mathematical modelling in the control of infectious diseases. Researchers in modelling have been working intensively around the effects of social distancing and travelling on the propagation of COVID-19 in the world, e.g. [2, 4, 13].

The World Health Organisation (WHO) has earmarked some diseases for eradication, see for instance [16]. In a region where a particular disease is well under control, it is possible that infected humans or vectors moving in from elsewhere can cause additional infections in the local population. Such additional infections can be disruptive to a local population in which there are systems in place to curb or eliminate the disease and where good progress is made in this regard. Examples of this phenomenon appear in [7, 11, 20]. It is important to identify and quantify the effects of such an infected inflow. In this paper we present a model for quantifying the effects of sporadic migration of infected individuals into and out of a region where there is good control and progress towards elimination. The model is of SEIR compartmental type.

There are numerous papers in the literature that present models for the population dynamics of infectious diseases. This includes models which accommodate the inflow of infected individuals. Examples of such papers with inflow of the infective are [1, 3, 15, 18, 21, 23]. The current paper makes another contribution in this regard. The objective is to model a combined population consisting of a *local population* together with *migrants* who stay for a short while. This seems to be the first model that provides for recovered migrants also to *depart* from the population at a constant rate. In particular this model is meant to be utilised for quantifying the effect of infected migrants on the local population.

We present a deterministic ordinary differential equations (ODE) model of four compartments for the combined population in Sect. 2. We prove that the inflow of infected migrants gives rise to positive threshold levels of the *E*-compartment and the *I*-compartment. Given the system of ODE, one can readily describe the dynamics of the migrant subpopulation. It is shown that the constant outflow of surviving migrants from the *R*-class is the correct number to balance the inflow, keeping the migrant population stable in the long run. In Sect. 3 we briefly do a similar investigation on an SIR model. In Sect. 4 we present an application to a measles problem, and in Sect. 5 we offer some concluding remarks.

## The model

The population comprising both *locals* and *migrants*, at any time *t*, consists of $N(t)$ individuals. We assume homogeneous mixing in the combined population. The population is divided into four compartments or classes. These are called the susceptible, exposed, infectious and removed classes. Their sizes, at any time *t*, are denoted by $S(t)$, $E(t)$, $I(t)$ and $R(t)$ respectively.

These variables are functions of time. All the parameters mentioned hereafter will be considered to be nonnegative constants. The symbols $K_{0}$, $K_{1}$, $K_{2}$ and $K_{3}$ denote constant recruitment into the population either through birth or through immigration, into the classes *S*, *E*, *I* and *R* respectively. The birth rate of the local population is $K_{0}+K_{3}$. Part of the newborns are vaccinated shortly after birth, causing flow into the class *R* at the rate $K_{3}$, while the remaining newborns move into the class *S* at the rate $K_{0}$. The inflow into the migrant subpopulation is at the rates $K_{1}$ and $K_{2}$ into the classes *E* and *I* respectively. There is an outflow of recovered migrants at the rate $K_{4}$.

Similarly as in [18], the force of infection in our model will be a function *f* which is twice differentiable, satisfying the following hypotheses:

1. $f(I)\geq 0$, $f(I)=0$ if and only if $I=0$.
2. $f '(I)\geq 0$ and $\beta := f '(0) >0$.
3. $f ''(I) \leq 0$.

An important consequence of hypotheses (H1)–(H3) on *f* is that, for each $I>0$, we have $If '(I) \leq f (I)$, see [18, Proposition 4.1]. Another inequality implied by this set of axioms is that, for every $I>0$, $f(I)/I \leq f '(0) =\beta $, i.e. $f(I) \leq \beta I$.

The rate of deaths due to the infectious disease in point is denoted by *δ*. Otherwise there is assumed to be a mortality rate *μ* in all the classes. The rate at which the latently infected become infectious is $\alpha _{1}$, and the rate at which the infectious recover is $\alpha _{2}$.

We introduce two more numbers:

1 $$\begin{aligned}& B_{1}= K_{1}/(\alpha _{1}+\mu ), \qquad B_{2}=(K_{2}+\alpha _{1} B_{1})/( \alpha _{2}+\mu +\delta ). \end{aligned}$$

The constant $K_{4}$ is the rate of outflow of migrants from the local population, and in particular, moving out of the *R*-class. We require $K_{4}$ to take the value

$$\begin{aligned}& K_{4}=\alpha _{2}B_{2}. \end{aligned}$$

The following system of equations is presented as our model.

### The system of ODE for the model

$$\begin{aligned}& S'(t) = K_{0}-\mu S(t)-S(t)f\bigl(I(t)\bigr), \\& E'(t) = K_{1}+ S(t)f\bigl(I(t)\bigr)-(\alpha _{1} +\mu )E(t), \\& I'(t) = K_{2}+\alpha _{1} E(t) -(\alpha _{2}+\delta +\mu )I(t), \\& R'(t) = K_{3} -K_{4} +\alpha _{2}I(t) -\mu R(t) \end{aligned}$$

with the initial conditions

$$\begin{aligned}& S(0) > 0,\qquad E(0)> B_{1},\qquad I(0) > B_{2},\qquad R(0) > 0. \end{aligned}$$

Let us write

2 $$\begin{aligned}& K=K_{0}+K_{1} +K_{2}+K_{3}-K_{4}. \end{aligned}$$

During the stay of the migrants in the local population, there will be mortalities, and these are accounted for in the constant $K_{4}$. One can readily check that $K_{4}\leq K_{1}+K_{2}$. This further implies in particular that $K>0$.

If we remove the fourth equation from the model system, then the remaining three equations will still follow the same trajectories. This approach is commonly followed in the literature, in particular when modelling viral infections, which are assumed to lead to permanent immunity and consequently no reinfection. See for instance [6, 17]. In that case the resulting SEI model is a special case of the model of Sigdel and McCluskey [18]. We shall refer to this subsystem of three equations as the SEI-special case. In particular, for the SEI-special case and with $K_{1}+K_{2}>0$, the unique endemic equilibrium point exists and is globally asymptotically stable [18].

We write $N(t)=S(t) + E(t) +I(t)+ R(t)$, and we introduce the sets

3 $$\begin{aligned}& \begin{aligned} &D_{0}=\bigl\{ x\in {\mathbb{R}}^{4} | x_{1}>0, x_{2}>B_{1}, x_{3} >B_{2}, x_{4}>0\bigr\} , \\ &D_{1}=\bigl\{ x\in {\mathbb{R}}^{4} | x_{1}>0, x_{2}>B_{1}, x_{3} >B_{2}, x_{4}>0, x_{1}+x_{2}+x_{3}+x_{4} \leq K/\mu \bigr\} . \end{aligned} \end{aligned}$$

It is important that solutions of our the model system are positive and bounded. We proceed with such investigations.

#### Proposition 2.1

*Consider a number* $t_{1}\in (0,\infty )$. *Suppose that* $X(t)$ *is a solution for system* (2.1) *with* $X(t) \in D_{0}$ *for all* $t\in [0,t_{1})$ *and* $N(0)< K/\mu $. *Then* $N(t) \leq K/\mu $ *for all* $t\in [0,t_{1})$.

#### Proof

$$ \frac{d(N(t)-K/\mu )}{dt} = -\mu \bigl(N(t)-K/\mu \bigr)-\delta I \leq - \mu \bigl(N(t)-K/\mu \bigr). $$

Therefore $N(0)< K/\mu $ implies that $N(t)< K/\mu $ for all $t\leq t_{1}$. □

The proof by contradiction that we use towards Theorem 2.2 has been popularly used, for instance, in [12] and [14].

#### Theorem 2.2

*Suppose that*, *for* $t\geq 0$, $X(t)$ *is a solution of* (2.1) *with* $X(0)\in D_{1}$. *Then* $X(t)\in D_{1}$ *for all* $t>0$.

#### Proof

The right-hand sides of the equations in the model system constitute a function of $(S,E,I,R)$ which is single-valued, continuous and continuously differentiable with respect to the variables *S*, *E*, *I* and *R*. Therefore, see [9] for instance, global solutions to the model system do exist and are unique.

The remainder of the proof is by contradiction. Thus let us suppose that the set

$$ Z:=\bigl\{ t>0: X(t) \notin D_{0}\bigr\} $$

is nonempty. Then *Z* has an infimum $z_{1}$, and $z_{1}$ is the smallest time-value for which $X(t)$ exits the set $D_{1}$. Let us write $J_{1}(t) = E(t)-B_{1}$ and $J_{2}(t)= I(t)-B_{2}$. Now we define a function $L:[0, \infty ) \to {\mathbb{R}}$ by the formula

$$ L(t)=\ln \biggl(\frac{K}{\mu S(t)} \biggr) + \ln \biggl( \frac{K}{\mu J_{1}(t)} \biggr) +\ln \biggl(\frac{K}{\mu J_{2}(t)} \biggr) + \ln \biggl(\frac{K}{\mu R(t)} \biggr). $$

Each of the four terms of the expression for $L(t)$ are nonnegative, and $\lim_{x\to 0^{+}}(\ln x) = +\infty $. Therefore,

4 $$ \lim_{t\to z_{1}^{-}} L(t) = \infty . $$

In what follows we derive a contradiction to equation (4). We note that, for any time $t_{1}>0$, $L(t_{1}) =\int _{0}^{t_{1}}L'(t)\,dt -L(0)$. Now we proceed to finding an upper bound for the image set $L[0, z_{1})$. We observe that, for any *t* with $0\leq t < z_{1}$,

$$\begin{aligned}& -\frac{S'(t)}{S(t)} = \frac{-1}{S(t)}\bigl[K_{0}-\mu S(t)-S(t)f \bigl(I(t)\bigr)\bigr] \leq \mu + f\bigl(I(t)\bigr) \leq \mu + \beta I \leq \mu + \beta \frac{K}{\mu } , \\& -\frac{J'_{1}(t)}{J_{1}(t)} = \frac{-1}{J_{1}(t)}\bigl[K_{1}+ S(t)f\bigl(I(t) \bigr)-( \alpha _{1} +\mu )E(t)\bigr] \\& \hphantom{-\frac{J'_{1}(t)}{J_{1}(t)}} \leq \frac{-1}{J_{1}(t)}\bigl[K_{1}-(\alpha _{1} +\mu ) \bigl(J_{1}(t)+B_{1}\bigr)\bigr] \\& \hphantom{-\frac{J'_{1}(t)}{J_{1}(t)}} = \frac{-1}{J_{1}(t)}\bigl[-(\alpha _{1} +\mu )J_{1}(t)\bigr] = \alpha _{1} +\mu, \\& -\frac{J'_{2}(t)}{J_{2}(t)} = \frac{-1}{J_{2}(t)}\bigl[K_{2}+\alpha _{1} E(t) -(\alpha _{2}+\delta +\mu )I(t)\bigr] \\& \hphantom{-\frac{J'_{2}(t)}{J_{2}(t)}}\leq \frac{-1}{J_{2}(t)}\bigl[K_{2}+\alpha _{1} B_{1} -(\alpha _{2}+ \delta +\mu )B_{2}-(\alpha _{2}+\delta +\mu )J_{2}(t)\bigr] = \alpha _{2}+ \delta +\mu ,\\& -\frac{R'(t)}{R(t)} = \frac{-1}{R(t)}\bigl[K_{3} +\alpha _{2}I(t) -\mu R(t) -K_{4}\bigr] \\& \hphantom{-\frac{R'(t)}{R(t)}} \leq \mu \frac{-1}{R(t)}\bigl[\alpha _{2}B_{2} -\mu R(t) -K_{4}\bigr] =\mu . \end{aligned}$$

Therefore we find

$$\begin{aligned}& L'(t)= -\frac{S'(t)}{S(t)} -\frac{J'_{1}(t)}{J_{1}(t)} - \frac{J'_{2}(t)}{J_{2}(t)} - \frac{R'(t)}{R(t)}\leq L_{0}, \end{aligned}$$

where $L_{0}=q (\mu +\beta K/\mu )+ {\alpha _{1} +\mu } +(\alpha _{2}+ \delta +\mu ) +\mu $. Consequently, we have

$$ \lim_{t\to z_{1}^{-}} L(t) =\lim_{t\to z_{1}^{-}} \int _{0}^{t} L'(v)\,dv \leq L_{0}z_{1}. $$

This contradicts the statement in equation (4). It means that such a finite number $z_{1}$ does not exist, and indeed that the variables are strictly positive over the entire interval of time $[0, \infty )$. □

#### Remark 2.3

- The system of ODE that describes the state $(E_{\mathrm{mi}}, I_{\mathrm{mi}}, R_{\mathrm{mi}})$ of the migrant subpopulation is as follows:
  $$\begin{aligned}& E'_{\mathrm{mi}}(t) = K_{1}-(\alpha _{1} + \mu )E_{\mathrm{mi}}(t), \\& I'_{\mathrm{mi}}(t) = K_{2}+\alpha _{1} E_{\mathrm{mi}}(t) -(\alpha _{2}+ \delta +\mu )I_{\mathrm{mi}}(t), \\& R'_{\mathrm{mi}}(t) = \alpha _{2}I_{\mathrm{mi}}(t) - \mu R_{\mathrm{mi}}(t) -K_{4} , \end{aligned}$$
  with the initial conditions $E_{\mathrm{mi}}(0) \geq B_{1}$, $I_{\mathrm{mi}}(0) \geq B_{2}$, $R_{\mathrm{mi}}(0) \geq 0$.
- A similar argument as in Theorem 2.2 can be followed to prove that any solution
  $$\bigl(E_{\mathrm{mi}}(t), I_{\mathrm{mi}}(t), R_{\mathrm{mi}}(t)\bigr) $$
  of the system in Remark 2.3(a) will be positive, provided that $E_{\mathrm{mi}}(0) > B_{1}$, $I_{\mathrm{mi}}(0) > B_{2}$, $R_{\mathrm{mi}}(0) > 0$.
- The unique equilibrium point has co-ordinates: $E^{*}_{\mathrm{mi}}(t)=B_{1}$, $I^{*}_{\mathrm{mi}}(t)=B_{2}$, $R^{*}_{\mathrm{mi}}(t)=0$.
- In model (2.1), if we replace $K_{4}$ with zero, i.e. if the only outflow from the model is death, then the minimum levels $B_{1}$ and $B_{2}$ would still hold. In fact one can investigate similar results in other models with the inflow of infection, provided that the initial values are above these minimum levels.
- If we take $E_{\mathrm{mi}}(0)=B_{1}$, $I_{\mathrm{mi}}(0)=B_{2}$, $R_{\mathrm{mi}}(0)=0$, then for every $t>0$ we shall have $E_{\mathrm{mi}}(t)=B_{1}$, $I_{\mathrm{mi}}(t)=B_{2}$ and, in particular, $R_{ \mathrm{mi}}(t)=0$. Therefore the choice of the constant outflow rate $K_{4}$ is the correct one to keep the migrant population size stable.

#### Proposition 2.4

*The unique equilibrium point of the model in Remark *2.3(a) *is globally asymptotically stable*.

#### Proof

Let us consider the function

$$\begin{aligned}& V_{0}(t)= \bigl(E_{\mathrm{mi}}(t)-B_{1}\bigr)+ \bigl(I_{\mathrm{mi}}(t)-B_{2}\bigr) +R_{\mathrm{mi}}(t). \end{aligned}$$

We can calculate its time derivative and simplify, eventually to obtain

$$\begin{aligned}& V_{0}'(t)= -\mu \bigl[E_{\mathrm{mi}}(t)-B_{1} \bigr]-(\mu +\delta )\bigl[I_{\mathrm{mi}}(t)-B_{2})\bigr] -\mu R_{\mathrm{mi}}(t). \end{aligned}$$

Thus $V_{0}(t)$ is a Lyapunov function, and it follows that the equilibrium point is globally asymptotically stable. □

If $K_{1}+K_{2}>0$, then there is no disease-free equilibrium point. Stability analysis of the SEI model is discussed in detail in [18].

If we consider the special case of model (2.1) for which $K_{1}=K_{2}=0$, then a disease-free equilibrium point $(K_{0}/\mu , 0, 0, K_{3}/\mu )$ does exist. In this case the basic reproduction number of the disease can be calculated, and it takes the form

$$ R_{0}= \frac{\alpha _{1} \beta K_{0}}{\mu (\alpha _{2}+\delta +\mu )(\mu +\alpha _{1})}. $$

Especially when working towards elimination of the disease, then it is also important to fully analyse this special case of zero influx of infected immigrants, see also [21].

#### Theorem 2.5

*Suppose that in the model system we have* $K_{1}=K_{2}=0$. *If* $R_{0}<1$, *then the disease*-*free equilibrium is globally asymptotically stable*.

#### Proof

If $R_{0}<1$, then the following inequality holds:

$$ \beta K_{0} - \biggl(\frac{\alpha _{1} + \mu }{\alpha _{1}} \biggr) (\alpha _{2}+ \delta +\mu ) < 0. $$

We can find and fix two numbers $\epsilon _{1} >0$ and $\epsilon _{2} >0$, which are sufficiently small, such that

$$ \beta K_{0} - \biggl( \frac{\alpha _{1} + \mu -\epsilon _{1}}{\alpha _{1}} \biggr) (\alpha _{2}+ \delta +\mu ) +\epsilon _{2}\alpha _{2} < 0. $$

Now we define a function

$$\begin{aligned}& L(t) = S(t)-\frac{K_{0}}{\mu }+\ln \biggl(\frac{K_{0}}{\mu S(t)} \biggr) + E(t) + \biggl(\frac{\alpha _{1} + \mu +\epsilon _{1}}{\alpha _{1}} \biggr)I(t) \\& \hphantom{L(t) =}{}+ \epsilon _{2} \biggl[ R(t)-\frac{K_{3}}{\mu }+\ln \biggl( \frac{K_{3}}{\mu R(t)} \biggr) \biggr]. \end{aligned}$$

This function is positive-definite with respect to the disease-free equilibrium point. The derivative $L'(t)$ can be written as

$$ L'(t) = -\mu S(t) \biggl(1-\frac{K_{0}}{\mu S(t)} \biggr)^{2} - \epsilon _{2} \mu R(t) \biggl(1-\frac{K_{3}}{\mu R(t)} \biggr)^{2} - \frac{K_{3}I(t)}{\mu R(t)}+ Q_{1}E(t) +Q_{2}I(t), $$

with

$$ Q_{1} = -(\alpha _{1}+\mu ) + \alpha _{1} \frac{\alpha _{1} + \mu -\epsilon _{1}}{\alpha _{1}} < -\epsilon _{1}< 0, $$

and

$$ Q_{2} = \frac{K_{0}f(I(t))}{\mu I} - \biggl( \frac{\alpha _{1} + \mu -\epsilon _{1}}{\alpha _{1}} \biggr) ( \alpha _{2}+ \delta +\mu ) +\epsilon _{2}\alpha _{2}. $$

From hypotheses (H1)–(H3) it follows that, for each $I>0$, we have $f(I)/I \leq \beta $. Therefore

$$ Q_{2}\leq \frac{\beta K_{0}}{\mu } - \biggl( \frac{\alpha _{1} + \mu -\epsilon _{1}}{\alpha _{1}} \biggr) ( \alpha _{2}+ \delta +\mu ) +\epsilon _{2}\alpha _{2} . $$

This means that $Q_{2}<0$. It follows that the function $L'(t)$ is negative-definite, hence that *L* is Lyapunov with respect to the disease-free equilibrium point. This completes the proof. □

In the application of this model, the basic reproduction number is quite significant. In particular, if $R_{0}<1$, then it means that the migrants are the only hurdle towards elimination. This would mean that when there are no more infected immigrants, there may still be new infection cases in the local population, but the number of active cases will decrease steadily towards elimination.

Let us write

$$\begin{aligned}& \mu _{0}= \alpha _{1} + \mu +\delta \quad \text{and} \quad \theta = 1+\mu / \alpha _{1}. \end{aligned}$$

#### Theorem 2.6

- *If* $K_{1}+K_{2}>0$, *then system* (2.1) *has a unique equilibrium point*.
- *If* $K_{1}+K_{2}=0$ *and* ${{\mathcal{R}}}>1$, *then system* (2.1) *has a unique endemic equilibrium point*.
- *In each of cases* (a) *and* (b), *the following equations are satisfied*:
  $$\begin{aligned}& \frac{K_{1}+\theta K_{2}}{\theta \mu _{0}}< I^{*}< \frac{K_{0}+K_{1}+\theta K_{2}}{\theta \mu _{0}}, \\& S^{*}=K_{0}/\bigl(\mu +f\bigl(I^{*}\bigr)\bigr), \\& E^{*}=\bigl[\mu _{0} I^{*}-K_{2}\bigr]/ \alpha _{1} >0, \\& R^{*}=\mu ^{-1}\bigl[\alpha _{2} I^{*} +K_{2}-K_{3}\bigr]>0. \end{aligned}$$

#### Proof

Let $M_{0}$ be the interval $M_{0}=[x_{0}, x_{1})$, where

$$ x_{0}=\frac{K_{1}+\theta K_{2}}{\theta \mu _{0}}, \qquad x_{1}= \frac{K_{0}+K_{1}+\theta K_{2}}{\theta \mu _{0}} , $$

and let us define a function

$$ g_{0}:M_{0}\to [0, \infty ), \qquad x\mapsto \frac{\mu [\mu _{0}\theta x -K_{1}-\theta K_{2}]}{K_{0}-[\mu _{0}\theta x -K_{1}-\theta K_{2}]}. $$

Then $g_{0}$ is continuous, $g_{0}(x_{0})=0$, and $g_{0}$ is unbounded. We further note that

$$ g_{0}'(x)= \frac{\mu \mu _{0}\theta K_{0}}{\{K_{0}-[\mu _{0}\theta x -K_{1}-\theta K_{2}]\}^{2}}>0 $$

and

$$ g_{0}''(x)= \frac{2\mu [\mu _{0}\theta ]^{2}K_{0}}{\{K_{0}-[\mu _{0}\theta x -K_{1}-\theta K_{2}]\}^{3}} . $$

Furthermore, we have $g_{0}''(x)>0$ for all $x\in M$. So, in fact, $g_{0}$ has the following properties:

- $g_{0}(x) \geq 0 $ for all $x\in M_{0}$,
- $g_{0}'(x) > 0$ for all $x\in M_{0}$,
- $g_{0}''(x) > 0$ for all $x\in M_{0}$,
- $g_{0}$ is unbounded.

Now we note that $S^{*}$ satisfies the following two equations:

$$\begin{aligned}& S^{*}=K_{0}/\bigl(\mu + f\bigl(I^{*}\bigr)\bigr) \quad \text{and}\quad S^{*}=\bigl[(\alpha _{1}+\mu ) E^{*}-K_{1}\bigr]/\alpha _{1} . \end{aligned}$$

From these two equations, by elimination of $S^{*}$ and expressing $E^{*}$ in terms of $I^{*}$, we obtain the equation

$$ f\bigl(I^{*}\bigr)=g_{0}\bigl(I^{*}\bigr). $$

(a) Note that $g_{0}(x_{0})=0$. If $K_{1}+ K2 >0$, then $x_{0}>0$, and therefore $f(x_{0})>0=g_{0}(x_{0})$. Therefore and due to the other properties of *f* and $g_{0}$, there exists $I^{*}\in M_{0}$ such that $f(I^{*})=g_{0}(I^{*})$. This proves (a).

(b) Suppose that $K_{1}+ K_{2} >0$. The inequality ${\mathcal{R}}>1$ implies that

$$ \alpha _{1} \beta K_{0}>\mu (\alpha _{2}+\delta +\mu ) (\mu +\alpha _{1}). $$

Next we observe that

$$ g'(0)= \frac{\mu \mu _{0}(\alpha _{1} +\mu )\alpha _{1}K_{0}}{[K_{0}\alpha _{1}]^{2}}= \frac{\mu \mu _{0}(\alpha _{1} +\mu )}{K_{0}\alpha _{1}} < \beta =f '(0). $$

Therefore, due to the properties of $g_{0}$ listed above, we conclude that there exists a unique number $I^{*} \in M_{0}$ for which $f(I^{*})=g_{0}(I^{*})$ and $I^{*}>0$. This completes the proof of (b).

(c) We have proved that $x_{0}< I^{*}<x_{1}$. The rest of (c) follows readily, and thus the proof of the theorem is complete. □

## SIR model

The SIR model in this section is very similar to the SEIR model. The population comprising both *locals* and *migrants*, at any time *t*, consists of $N(t)$ individuals. The constants $K_{0}$, $K_{1}$ and $K_{2}$ denote recruitment rates into the classes *S*, *I* and *R* respectively. The birth rate of the local population is $K_{0}+K_{2}$. Part of the newborns are vaccinated shortly after birth, giving rise to the inflow at the rate $K_{2}$ into class *R*, while the remaining newborns move into class *S* at the rate $K_{0}$. The inflow into the migrant subpopulation is at the rate $K_{1}$ and all of these individuals are assumed to be infected. Again as in the SEIR case, we consider the force of infection to be a function *f* which is twice differentiable, satisfying hypotheses (H1), (H2) and (H3). The rate at which the infectious recover is *α*. The rate of deaths due to the specific infectious disease in point is denoted by *δ*. Otherwise there is a mortality rate *μ* in each of the classes.

We introduce the numbers *B* and *K* below, and we assume as fixed that $K_{3}$ takes the specified value:

5 $$\begin{aligned}& B= K_{1}/(\alpha +\mu +\delta ),\qquad K=K_{0}+K_{1} +K_{2}-K_{3}\quad \text{and}\quad K_{3}=\alpha B. \end{aligned}$$

### The system of ODE

$$\begin{aligned}& S'(t) = K_{0}-\mu S(t)- S(t)f\bigl(I(t)\bigr), \\& I'(t) = K_{1}+ S(t)f\bigl(I(t)\bigr)-(\alpha +\mu + \delta )I(t), \\& R'(t) = K_{2}+ \alpha I(t) -\mu R(t) -K_{3} \end{aligned}$$

with the initial conditions $S(0) > 0$, $I(0) >B$, $R(0) >0$.

Let $D_{2}$ be the set

$$D_{2}=\bigl\{ x\in {\mathbb{R}}^{3} | x_{1}>0, x_{2}>B, x_{3} >0, x_{1}+x_{2}+x_{3} \leq K/\mu \bigr\} . $$

For the special case having $K_{1}=0$, there is a disease-free equilibrium $(K_{0}/\mu , 0, K_{2}/\mu )$. Also for this special case we can calculate the basic reproduction number as

$$ {\mathcal{R}}= \frac{ \beta K_{0}}{\mu (\alpha +\delta +\mu )}. $$

#### Theorem 3.1

*Suppose that* $X(t)$ *is a solution for the SIR model system with* $X(0) \in D_{2}$. *Then* $X(t) \in D_{2}$ *for all* $t > 0$.

A proof for Theorem 3.1 can be obtained along the same lines as those of Proposition 2.1 and Theorem 2.2, and we omit the proof.

#### Remark 3.2

- The system of ODE that describes the state $(I_{\mathrm{mi}}, R_{\mathrm{mi}})$ of the migrant subpopulation is as follows:
  $$\begin{aligned}& I'_{\mathrm{mi}}(t) = K_{1}-(\alpha +\mu +\delta )I_{\mathrm{mi}}(t), \\& R'_{\mathrm{mi}}(t) = K_{2}-K_{3} +\alpha I_{\mathrm{mi}}(t) -\mu R_{\mathrm{mi}}(t) \end{aligned}$$
  with the initial conditions $I_{\mathrm{mi}}(0) \geq B$, $R_{\mathrm{mi}}(0) \ge 0$.
- Solutions $(I_{\mathrm{mi}}(t), R_{\mathrm{mi}}(t))$ of this system can be proved to be positive provided that $I_{\mathrm{mi}}(0) > B$, $R_{\mathrm{mi}}(0) > 0$.
- The unique endemic equilibrium point has coordinates $I^{*}_{\mathrm{mi}}(t)=B$, $R^{*}_{\mathrm{mi}}(t)=0$ and is globally asymptotically stable.
- From (c) we deduce that the choice of $K_{3}$ is the correct one to keep the migrant population size stable.

#### Notation 3.3

Henceforth in this section, for convenience we shall use the notation

$$ \mu _{1} = \mu +\delta + \alpha . $$

#### Theorem 3.4

- *If* $K_{1}>0$, *then the SIR model system has a unique equilibrium point*.
- *If* $K_{1}=0$ *and* ${\mathcal{R}}>1$, *then the SIR model system has a unique endemic equilibrium point*.
- *In each of these cases* (a) *and* (b),
  $$\begin{aligned}& \frac{K_{1}}{\mu _{1}} < I^{*}< \frac{K_{0}+K_{1}}{\mu _{1}}, \\& S^{*}=K_{0}/\bigl(\mu +f\bigl(I^{*}\bigr)\bigr), \\& R^{*}=\mu ^{-1}\bigl[\alpha I^{*} +K_{2}-K_{3}\bigr]. \end{aligned}$$

#### Proof

Let $M_{1}$ be the interval

$$ M= \bigg[\frac{K_{1}}{\mu _{1}}, \frac{K_{0}+K_{1}}{\mu _{1}} \bigg). $$

Associated with the parameter $K_{1}$, let us define a function

$$ g_{1}:M_{1}\to [0, \infty ), \qquad x\mapsto \frac{\mu (\mu _{1}x-K_{1})}{K_{0}+K_{1}-\mu _{1}x}. $$

Then in particular $g_{1}$ is continuous, $g_{1}(\frac{K_{1}}{\mu _{1}})=0$ and $g_{1}$ is unbounded. We further note that

$$ g_{1}'(x)=\frac{\mu \mu _{1}K_{0}}{[K_{0}+K_{1}-\mu _{1}x]^{2}}>0 $$

and

$$ g_{1}''(x)=\frac{2\mu \mu _{1}^{2}K_{0}}{[K_{0}+K_{1}-\mu _{1}x]^{3}} . $$

For $x\in M_{1}$, we have $x<(K_{0}+K_{1})/\mu _{1}$. Therefore $g_{1}''(x)>0$ for all $x\in M_{1}$. So, in fact, $g_{1}$ has the following properties:

- $g_{1}(x) \geq 0 $ for all $x\in M_{1}$,
- $g_{1}'(x) > 0$ for all $x\in M_{1}$,
- $g_{1}''(x) > 0$ for all $x\in M_{1}$,
- $g_{1}$ is unbounded.

Now we note that there are the following expressions for $S^{*}$:

$$\begin{aligned}& S^{*}=K_{0}/\bigl(\mu + f\bigl(I^{*}\bigr)\bigr) \quad \text{and}\quad S^{*}=\bigl(\mu _{1} I^{*} - K_{1}\bigr)/f\bigl(I^{*}\bigr). \end{aligned}$$

Elimination of $S^{*}$ between these two equations leads to the equation

$$ f\bigl(I^{*}\bigr)=g_{1}\bigl(I^{*}\bigr). $$

(a) Consider the case $K_{1}>0$. Given *g* as above, note that $g_{1}(K_{1}/ \mu _{1})=0 < f(K_{1}/\mu _{1})$. This, together with *f* being nonnegative and $f ''(I)\ge 0$, implies that there exists a unique value $I^{*} \in M_{1}$ for which $f(I^{*})=g_{1}(I^{*})$. This proves (a).

(b) Let us assume that $K_{1}=0$ and ${\mathcal{R}}>1$. The inequality ${\mathcal{R}}>1$ implies that $\beta K_{0}>\mu \mu _{1}$. Next we observe that

$$ g_{1}'(0)=\frac{\mu \mu _{1}K_{0}}{K_{0}^{2}}= \frac{\mu \mu _{1}}{K_{0}}< \beta =f '(0). $$

Again in view of the properties of $g_{1}$ listed above, we conclude that there exists a unique value $I^{*} \in M_{1}$ for which $f(I^{*})=g_{1}(I^{*})$ and $I^{*}>0$. This completes the proof of (b).

(c) This is clear. □

#### Theorem 3.5

*The endemic equilibrium point of the SIR model system is locally asymptotically stable*.

#### Proof

We calculate the Jacobian *J* of the system at the equilibrium point, and then we obtain its characteristic equation to be as follows:

$$ (-\mu -\lambda )\bigl[\lambda ^{2} + b_{2}\lambda + c_{2}\bigr] =0, $$

with $b_{2}$ and $c_{2}$ as below:

$$\begin{aligned}& b_{2} =\mu _{1}-S^{*}f ' \bigl(I^{*}\bigr) +\mu +f\bigl(I^{*}\bigr), \\& c_{2} = \mu \bigl[\mu _{1}-S^{*}f ' \bigl(I^{*}\bigr)\bigr] + \mu _{1}f\bigl(I^{*} \bigr). \end{aligned}$$

Hypotheses (H1), (H2) and (H3) on *f* ensure that, for every $I>0$, we have

$$ If '(I)\leq f(I). $$

Thus we can deduce the following:

$$ \mu _{1}-S^{*}f '\bigl(I^{*}\bigr)= \frac{\mu _{1}I^{*}-S^{*}I^{*}f '(I^{*})}{I^{*}}\geq \frac{\mu _{1}I^{*}-S^{*}f(I^{*})}{I^{*}}=\frac{K_{1}}{I^{*}}. $$

Therefore $\mu _{1}-S^{*}f '(I^{*})\geq 0$. Hence $b_{2}>0$ and $c_{2}>0$, and consequently all the characteristic roots have negative real parts. This concludes the proof. □

## Application to measles

Measles, a highly contagious viral disease, has been the cause of millions of deaths annually around the globe before vaccination started in 1963 [22]. Even now, with a safe and effective vaccine available, the prevalence and annual mortality due to measles is still startlingly high, with 140,000 mortalities in 2018. In 2019, more than 500,000 confirmed cases occurred globally amid devastating outbreaks [5]. Nevertheless, measles elimination programmes are in place in some countries, and in populations which are making progress it is especially important to be wary of infected people from elsewhere entering into the population. In what follows we consider measles in South Africa.

For this application we utilise the SEIR model system, and for the force of infection *f* we choose the specific form

$$ f(I)=\frac{\beta I}{1+bI} \quad \text{for some constant } b\geq 0. $$

For our simulations we use the Euler finite difference method. This methodology is still appropriate and a popular choice for modelling disease dynamics with ODEs, see for instance [4]. Most of the parameter values are either derived or taken directly from the literature as indicated in Table 1. The contact rate is fitted to data on South Africa.

**Table 1:** Numerical values of parameters

Param.	Description	Numerical value (per week)	Reference/comment
*μ*	mortality rate, excluding death directly due to measles	0.000298/week per week	[19]
*δ*	rate of human deaths due to measles	0.019/week	[5]
$\alpha _{1}$	transfer rate from *E*-class to *I*-class	1.05/week	[10]
$\alpha _{2}$	transfer rate from *I*-class to *R*-class (recovery rate)	0.049/week	[10]
$P_{0}$	local population size when disease-free	57.79 million	[24]
$K_{0}$	internal growth rate in class *S*	0.25 $\mu P_{0}$/week	[8]
$K_{1}$	rate of inflow of latently infected migrants	0–15 p.a.	variable
$K_{2}$	rate of inflow of infectious migrants	0	nominal
$K_{3}$	rate of vaccination of newborns	0.75 $\mu P_{0}$/week	[8]
*b*	saturation constant	0.01/week	estimated
*β*	contact rate at disease-free state	$1.409/P_{0}$ per week	fitted from [8]

The local population in 2018 was of size 58.49 million [24]. During 2018 there were 69 cases in South Africa [8]. We use this to estimate the contact rate. We take the initial point to be $E(0)= 0.5146$ and $I(0)=0.3229$, while $S(0) +R(0)= 57.789$ million, with $R(0)$ and $S(0)$ split according to the vaccination levels. Using the model, we calculate the number of new local measles cases which are caused if over a period of one year we have different levels of influx of latently measles infected people. Firstly we consider a vaccination rate of 75%, and then we repeat these calculations for a different level of vaccination, 95%.

**Figure 1:**
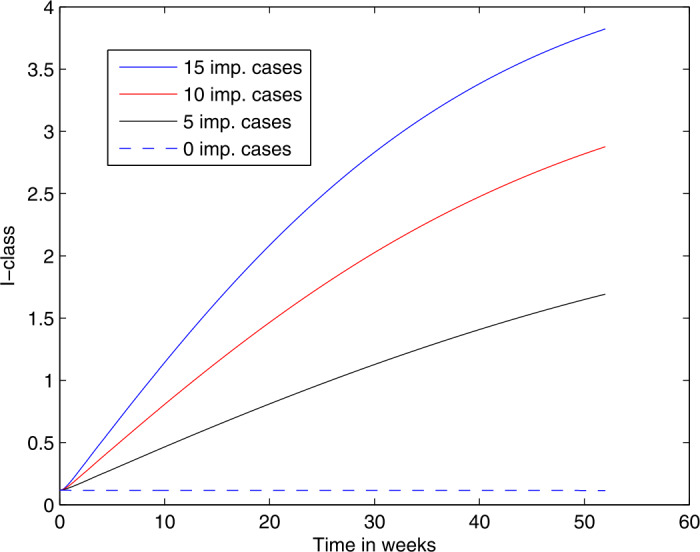
The trajectories of the active local infections *I* for different levels of annual imported infected cases, given that 75% of newborns are being vaccinated

**Figure 2:**
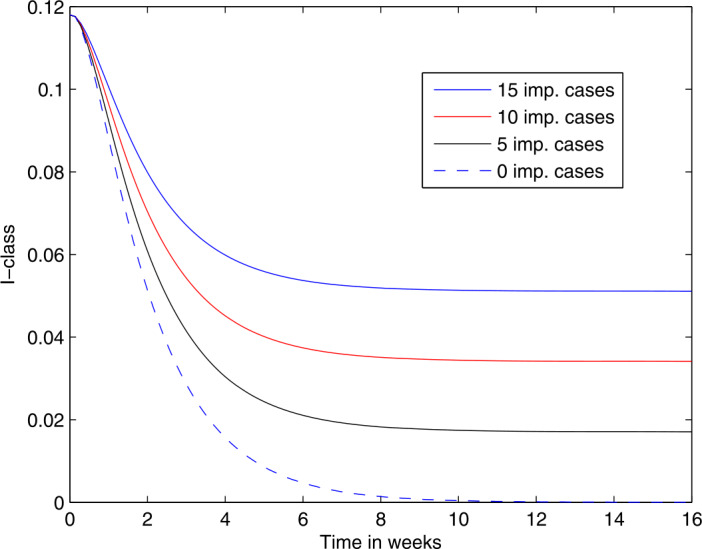
The trajectories over a period of 16 weeks, of the active local infections *I* for different levels of annual imported infected cases. It is assumed that 95% of newborns are being vaccinated

The curves in Fig. 1 are increasing, revealing the extent to which an inflow of infected individuals can cause a surge in local infections. In Fig. 2, the decreasing curves show firstly how a higher vaccination coverage (95% versus 75%) will lead to much lower prevalence of measles. Note that the initial state for these computations is the equilibrium value corresponding to a lower vaccination coverage of (75%), hence the curves dropping when the vaccination rate increases. Then secondly, the curves show the effects of different intensities of influx of infectives, on the prevalence of measles. Table 2 shows the number of new measles cases for a given influx rate of infected migrants and for different levels of vaccination. The difference between 95% vaccination as compared to the current 75% of population vaccinated is dramatic, and this further underlines the call of the NICD for 95% of newborns to be vaccinated.

**Table 2:** Model output for infection cases

Total number of imported cases	Vacc. 75% new cases	Vacc. 95% new cases
0	59	1.8
5	505	10.3
10	887	18.8
15	1223	27.2

## Conclusion

In the literature up to now, a population with two distinct components has been approached by way of a two-group model. In our situation we follow a much simpler single group approach. In conventional models with inflow of infected, the immigrants usually become part of the one single population, whereas in our current model we have found a way to quantify the rate of removal of migrants out of the population after a short visit. The new SEIR model with migration was shown in detail to comply to the necessary conventional requirements including positivity and boundedness of solutions, and stability of equilibrium points. We have also briefly explained an SIR model with migration to test the effect of the importation or inflow of infections on the local population in point.

The SEIR model has been applied in a simple manner to a problem of imported measles infection. If more information is available, such as the initial state of the local population, we will be able to achieve improved accuracy in calculation of future projections. However, even only working around an equilibrium point as we did already gives a good idea of what to expect. This methodology shows promise of versatile applicability.

## Data Availability

All the data used in this study were obtained from the literature.
